# AID-ing Signaling in *Toxoplasma gondii*

**DOI:** 10.1128/mBio.01076-17

**Published:** 2017-07-25

**Authors:** Kami Kim

**Affiliations:** Departments of Medicine, Pathology and Microbiology & Immunology, Albert Einstein College of Medicine, Bronx, New York, USA

**Keywords:** *Toxoplasma*, cell signaling, gene regulation, genetics, kinases

## Abstract

The cyclic GMP-dependent protein kinase (PKG) of apicomplexan parasites is essential for secretion of micronemes and host cell invasion and egress. Both kinase specificity and localization can determine which substrates are phosphorylated. The functions of plasma membrane and cytosolic PKG isoforms of *Toxoplasma gondii* were unknown because of difficulties precisely manipulating expression of essential genes. Brown et al. (K. M. Brown, S. Long, and L. D. Sibley, mBio 8:e00375-17, https://doi.org/10.1128/mBio.00375-17) adapted the auxin-inducible degron (AID) system for conditional expression of *T. gondii* proteins. AID, in combination with clustered regularly interspaced short palindromic repeat (CRISPR)-Cas9 gene editing, facilitated creation of a panel of PKG mutants to demonstrate that the membrane association via acylation of PKG is critical for its essential functions in tachyzoites. The cytosolic form of PKG is not sufficient for viability and is dispensable. These studies illuminate a critical role for targeting of kinase complexes for parasite viability. The AID system enables rapid, conditional regulation of protein expression that expands the molecular toolbox of *T. gondii*.

## COMMENTARY

The Apicomplexa are obligate intracellular parasites that include important human and veterinary parasites, including *Plasmodium* species, *Cryptosporidium* species, and *Toxoplasma gondii*. Among the Apicomplexa, *T. gondii* is the most genetically tractable and has been a used as a model organism to discover many aspects of conserved parasite biology, including understanding parasite egress and invasion pathways.

The era of chemical biology in Apicomplexa was ushered in by elegant work by Gurnett et al. (1) and Donald et al. (2), who used a novel kinase inhibitor, compound 1, to identify cyclic GMP-dependent protein kinase (PKG) as an essential gene regulating several aspects of apicomplexan parasite biology (3). PKG has a critical role in invasion and microneme secretion for both *T. gondii* and *Plasmodium* species (4). Subsequent studies by several groups identified calcium-dependent protein kinases (CDPKs) as regulators of similar pathways, and both PKG and CDPKs have subsequently emerged as important novel targets for antiparasitic agents specific for the Apicomplexa. Further studies have also revealed unique stage-specific roles of these kinases, but fully understanding several aspects of their biological function has been thwarted by their essential functions and by the fact that Apicomplexa are obligate intracellular parasites that cannot be cultivated axenically.

*T. gondii* PKG is associated with the cell periphery, and PKG molecular interaction with membranes is based upon acylation by myristate followed by acylation with palmitate (5). In coccidian parasites, including *T. gondii* and *Eimeria* and *Neospora* spp., PKG has two isoforms that differ only in the presence or absence of the N-terminal acylation sequence that results in plasma membrane localization. Initial studies suggested that the two forms were functionally redundant (5), but these studies were performed using genetic techniques that relied upon overexpression of a second copy of the gene; at the time, precise conditional expression of PKG was not technically feasible. Although PKG is essential in Apicomplexa, many species have only a single form of PKG, suggesting that each PKG isoform has a unique role in tissue-cyst forming coccidia.

Since the original studies investigating PKG biology were reported, the repertoire of tools available for manipulation of gene expression in the Apicomplexa has expanded dramatically. Clustered regularly interspaced short palindromic repeat (CRISPR)-Cas9 gene-editing technology has revolutionized the field and enabled rapid generation of precise mutations, and now it is possible to create mutants that express a target protein in the correct genomic context and create transgenic parasites in nearly any genetic background. For essential genes, conditional expression is required to understand the molecular function of genes. Both tetracycline-regulated transcriptional systems and posttranslational conditional expression systems using the FK506-binding protein (FKBP)-inducible degradation domain (DD) have been developed for *T. gondii*, but induction of these systems is accompanied by a lag or slow depletion of protein levels. In addition, because the DD-stabilizing agent Shield must be continuously applied to stabilize proteins, the DD system can be expensive and cumbersome.

The auxin-inducible degron (AID) system has improved kinetics and responsiveness in *T. gondii* compared to the DD system (15 to 30 min versus hours) (6). Coupled with CRISPR-Cas9 gene-editing techniques, multiple mutations can be generated quickly. The AID system is based upon the conserved SCF ubiquitin ligase complex that consists of Skp1, Cullin1, and an F box protein (7). The F box recruits specific substrates for degradation by the proteosomal system. Auxin stabilizes the interaction of the auxin receptor TIR1 (an F box protein) with proteins that encode an auxin-inducible degron (AID) motif (7). TIR1 is specific to plants, but conservation of the SCF complex allows TIR1 to form a functional degradation complex in nonplant cells (7). Thus, proteins expressing an AID motif stably interact with the SCF complex via TIR1, are polyubiquinated by endogenous SCF, and are degraded by the proteasome.

To use AID, Brown et al. created a stable cell line in the RH strain background expressing a *T. gondii* codon-optimized TIR1 (6). Expression of yellow fluorescent protein (YFP) control protein with a mini-AID tag (68 amino acids), containing a minimal AID motif (mAID), in the TIR1 strain resulted in depletion of protein 15 min after auxin addition. Pretreatment with proteasome inhibitors resulted in stabilization of the YFP-mAID, confirming the role of the proteasome in protein depletion. Expression of CDPK1-mAID-3HA (an essential gene with a well-characterized function) and PKG-mAID-3HA was responsive to auxin and prevented lytic infection of tachyzoites, as measured by plaque assays. For PKG-mAID-3HA parasites, both the membrane-associated and cytosolic PKG forms were detectable. Auxin had no effect on parasite replication, suggesting a role for PKG in motility, egress, or invasion. Complementation of PKG-mAID-3HA parasites with different PKG mutants revealed that the membrane-associated form was sufficient for critical functions, including microneme secretion and invasion as well as parasite egress. Parasite lines complemented with cytosolic forms of PKG were unable to make plaques and had reduced capacity for microneme secretion, invasion, and egress. The PKG cytosolic form was dispensable for parasite growth, indicating that, in tachyzoites, only the membrane-associated form of PKG is critical for parasite viability (6).

Ubiquitination is common among *T. gondii* proteins (8), and although few obvious F box proteins are found in the *T. gondii* genome, SKP1 and cullins are conserved. The utility of the AID system provides functional evidence that the mechanics of the SCF ubiquitin ligase machinery are conserved in *T. gondii*, as in other eukaryotes, including *Plasmodium* species.

A number of *T. gondii* signaling molecules are predicted to interact with membranes via acylation domains. Conventional scaffolding proteins that regulate signaling, such as AKAPs (cyclic AMP [cAMP]-regulated kinase-associated proteins), have not been described, and many apicomplexan signaling proteins, including PKG and CDPKs, are predicted to contain N-terminal acylation motifs. As reported for PKG, acylation usually requires N-terminal myristoylation followed by palmitoylation. Since acylation and proper targeting are critical for the essential functions of PKG, development of compounds that target membrane trafficking of PKG may pose an alternative strategy for development of antiparasitic agents. Protein acylation also is common in the Kinetoplastida (9), and thus acylation enzymes (N-myristoyl transferases or palmitoyl transferases) may prove to be potential general targets for treatment of parasitic infections.

In the tissue-cyst forming coccidian Apicomplexa (*T. gondii*, *Hammondia*, *Neospora*, and *Eimeria*), both the membrane and cytosolic PKG forms are conserved, suggesting that the cytosolic form of PKG has an important function in another aspect of parasite biology. In addition to its roles in parasite invasion and egress, *Plasmodium falciparum* PKG (PfPKG) has an essential role in gametocyte development (4). By analogy, a role for cytosolic forms of *T. gondii* PKG (TgPKG) in other developmental stages is possible. The TIR1 stable strain was made in the RH background, which is unable to differentiate to bradyzoites or to complete the full life cycle in feline intestines. Assuming that TIR1 strains are readily made in other strains of *T. gondii*, the AID system might clarify if PKG has another role in other developmental stages that was not evident under the tachyzoite experimental conditions tested. In addition, application of AID may enable dissection of the molecular mechanisms of complex cell signaling pathways in *T. gondii* and elucidate the molecular mechanisms and functional interplay of potentially redundant PKG and CDPKs in different stages of *T. gondii* development.

The AID system was also adapted to the *Plasmodium berghei* rodent malaria model, where it led to protein depletion in 45 min (10), demonstrating an essential role for calcineurin (PbCn). Philip and Waters administered auxin to synchronized, *in vitro*-cultivated, PbCnA-AID parasites at specific points in *Plasmodium* development and performed a battery of functional and morphological assays (10). These assays demonstrated a role for calcineurin in erythrocyte attachment and invasion, male gametocytogenesis, and fertilization, as well as ookinete-to-oocyst and sporozoite-to-liver-stage transitions (10).

Recently, in a major breakthrough, genetic modification of *Cryptosporidium parvum* was achieved using CRISPR-Cas9 in a mouse model (11). Neither *Cryptosporidium* nor *T. gondii* sexual stages can be propagated in culture, but several groups are working to propagate these parasites in organoid models. AID, adapted for use in mouse or organoid models, may also be a useful tool for regulated expression of genes in apicomplexan species or life cycle stages that were formerly inaccessible to experimental manipulation.
